# Risk of malignant lymphoma associated with human herpesvirus-8: a case–control study in Spain

**DOI:** 10.1038/sj.bjc.6601858

**Published:** 2004-05-04

**Authors:** S de Sanjosé, J J Goedert, V Marshall, C Bellas, Y Benavente, R Bosch, A Domingo, A Fernandez de Sevilla, O Servitje, D Whitby

**Affiliations:** 1 Servei d'Epidemiologia i Registre del Cancer, Institut Catala d'Oncologia, Barcelona, Spain; 2 Viral Epidemiology Branch, National Cancer Institute, Bethesda, MD, USA; 3 Viral Epidemiology Section, AVP, SAIC-Frederick, National Cancer Institute-Frederick, Frederick, MD, USA; 4 Patologia, Ramon y Cajal, Madrid, Spain; 5 Patologia, Hospital Verge de la Cinta, Tortosa, Spain; 6 Hematology, Hospital de Bellvitge, Barcelona, Spain; 7 Hematologia Oncologica, Institut Catala d'Oncologia, Barcelona, Spain; 8 Dermatology, Ciutat Sanitaria and Universitaria de Bellvitge, Barcelona, Spain

**Keywords:** lymphoma, herpesvirus-8, case–control study

## Abstract

No overall increased risk of lymphoma associated with antibodies to human herpesvirus-8 was found in 526 lymphomas and 599 controls (odds ratio (OR)=1.04, 95% confidence interval (CI)=0.62–1.75); significant increases were noted for 19 lymphoplasmacytic lymphomas (OR=4.47, 95% CI=1.34–14.85) and nine low-grade lymphoma/lymphoma B-cell NOS (OR=5.82, 95% CI=1.07–31.73).

Kaposi's sarcoma (KS)-associated herpesvirus, also known as human herpesvirus-8 (HHV-8), has been shown to be causally associated with KS, primary effusion lymphoma (PEL) and multicentric Castleman's disease (MCD) (Chang *et al*, 1994; Soulier *et al*, 1995; Cesarman *et al*, 1996). The three conditions are increased in immunocompromised states, as in human immunodeficiency virus (HIV) infection. The association of HHV-8 with other lymphoproliferative disorders in non-HIV-infected subjects remains controversial (Mikala *et al*, 1999). In the absence of HIV, HHV-8 DNA has been detected in T-cell PEL cells (Lechapt-Zalcman *et al*, 2001) and in lymphoma cells with plasmacytic differentiation, but not in cutaneous T- and B-cell lymphoma (Dupin *et al*, 1997) or in mycosis fungoides (Henghold *et al*, 1997).

A systematic serological evaluation of HHV-8 in HIV seronegative cancer patients failed to identify a significantly increased prevalence among patients with lymphoid neoplasms (Sitas *et al*, 1999). A similar study design in Uganda identified a slightly higher HHV-8 prevalence among patients with non-Hodgkin lymphoma (61%) and Hodgkin lymphoma (61%) as compared to the control population (50%). Differences were not statistically significant (Newton *et al*, 2003).

In this study, in Spain, we evaluated the association between HHV-8 infection and malignant lymphoma.

## MATERIALS AND METHODS

The study subjects were recruited at four centres in Spain: Barcelona, two in Tarragona (Tortosa and Reus) and Madrid. Cases were consecutive patients newly diagnosed with a lymphoid malignancy between 1998 and 2002 and categorised according to the WHO Classification for Neoplastic Diseases of the Lymphoid Tissues (Jaffe *et al*, 2003). Controls were randomly selected from the hospital wards and outpatient clinics daily lists and synchronically identified with the cases. Controls were frequency matched to the cases by age, sex and study centre. Subjects with cancer, organ transplant and/or systemic infection as main diagnosis were not eligible as controls.

All included subjects were interviewed on demographic, medical and family history, and environmental exposures. Cases and controls provided a blood sample. Informed consent was obtained from all subjects prior to enrolment, and the Institutional Review Boards of the participating centres approved the study.

Of 700 eligible cases, 526 (75%) were included in the study, 28 refused to participate, 25 died before the interview, 116 did not provide a blood sample and five cases had no interview. Of 655 eligible controls, 599 (91.6%) were included in the study, 23 refused to participate and 33 did not provide a blood sample. Further details of the study have been described elsewhere (de Sanjose *et al*, 2004).

### HHV-8 antibody and HIV detection

Antibodies against the lytic antigent K8.1 were tested using a enzyme-linked immunoassay (ELISA) as described previously (de Sanjose *et al*, 2002). Samples with optical densities (OD) below 1 were considered to be negative. Antibodies against the open-reading frame 73 (LANA) were tested by a similar ELISA using full-length baculo expressed LANA as antigen and serum diluted 1 : 100. Optical densities values below 0.8 were considered to be negative. All the sera were tested blind to the disease status.

HIV infection status was determined by testing the sera with a licensed commercial ELISA (Abbot Diagnostics, North Chicago, IL, USA). All positive subjects were confirmed with Western blot.

### HHV-8 quantitative real-time PCR

Peripheral blood mononuclear cells (PBMC) were tested for HHV-8 DNA by quantitative PCR in all subjects considered to be seropositive for either anti-K8.1 or anti-LANA as described previously (de Sanjose *et al*, 2002). DNA quality and cell quantitation was determined using real-time PCR for endogenous retrovirus 3 (Yuan *et al*, 2001).

### Statistical analyses

Unconditional logistic regression was used to estimate the odds ratios (OR) and 95% confidence interval (95% CI) in order to measure the association between specific variables and the risk of lymphoma. Questionnaire variables were explored for their association with HHV-8 or with case–control status at *P*<0.10 and considered for inclusion in the regression model. The contribution to the models by other potential confounding variables was tested by means of the likelihood ratio test.

## RESULTS

The study population consisted of 526 lymphoma cases and 599 controls. The average age at entry was 59.7 years among cases and 58.0 years among controls.

No differences were observed in the distribution of cases and controls in relation to age, sex, recruitment area, educational level and history of blood transfusion (Table 1

**Table 1:** Distribution of cases and controls by sociodemographic characteristics

	**Controls**	**Lymphoma cases**
	**Number (%)**	**Number (%)**
Total	600 (100)	529 (100)
*Age (years)*		
<43	130 (21.7)	95 (18.0)
43–56	123 (20.5)	106 (20.0)
57–67	124 (20.7)	106 (20.6)
68–74	114 (19)	124 (23.6)
>74	108 (18)	95 (18.1)
		
*Sex*		
Males	312 (52.1)	287 (54.6)
Females	287 (47.9)	239 (45.4)
		
*Recruitment area*		
Barcelona	500 (83.3)	411 (78.1)
Madrid	55 (9.2)	68 (12.9)
Tarragona	44 (7.3)	47 (8.9)
		
*Educational level attained*		
Primary	237 (39.6)	212 (40.3)
Secondary	60 (10.0)	39 (7.4)
Higher school	17 (2.8)	20 (3.8)
University	41 (6.8)	44 (8.4)
Other	38 (6.3)	33 (6.3)
No degree	138 (23.0)	110 (20.9)
Never school	61 (10.2)	67 (12.7)
		
History of blood transfusion	160 (26.7)	129 (24.5)

). Of all the items explored, low educational level was significantly associated with higher prevalence of HHV-8 among controls, but the educational level did not modify the overall risk estimates (data not shown).

HIV infection was detected in 17 cases and one control, while eight cases had a history of organ transplant. HHV-8 was detected in four of these subjects (15.4%). These patients are excluded in the following results if not otherwise specified.

In all, 32 controls (5.4%) and 29 cases (5.8%) were HHV-8 positive (Table 2

**Table 2:** OR for HHV-8 (K8.1 or LANA) detection among lymphoma categories and age–sex matched controls

	**Total, *N***	**K8.1, *N*+ (%)**	**LANA, *N*+ (%)**	**K8.1 or LANA, *N*+ (%)**	**OR^b^ (95% CI)**
Controls	598	24 (4)	17 (2.8)	32 (5.4)	Ref
					
All lymphoid neoplasm	501	43 (8.6)	16 (3.2)	29 (5.8)	1.04 (0.62–1.75)
					
B-cell lymphomas	464	22 (4.7)	15 (3.2)	27 (5.8)	1.03 (0.60–1.76)
					
Chronic lymphocytic leukaemia	115	6 (5.2)	3 (2.6)	7 (6.1)	1.16 (0.48–2.79)
Lymphoplasmacytic lymphoma	19	4 (21.1)	1 (5.3)	4 (21.1)	4.47(1.34–14.85)
Marginal zone	25	0 (0)	0 (0)	0 (0)	NA
Splenic marginal zone	26	3 (11.5)	1 (3.8)	3 (11.5)	2.50 (0.68–9.14)
Plasma cell myeloma	70	2 (2.9)	2 (2.9)	2 (2.9)	0.53 (0.12–2.30)
Follicular lymphoma	37	1 (2.7)	0 (0)	1 (2.7)	0.46 (0.06–3.57)
Diffuse large B cell	82	3 (3.7)	3 (3.7)	5 (6.1)	1.08 (0.40–2.89)
Low-grade B and lymphoma B nos.	9	2 (22.2)	2 (22.2)	2 (22.2)	5.82 (1.07–31.73)
Other B-cell lymphoma^c^	25	0 (0)	0 (0)	0 (0)	NA
Hodgkin lymphoma	56	1 (1.8)	2 (3.6)	3 (5.4)	0.97 (0.27–3.43)
					
T-cell lymphomas	37	1 (2.7)	1 (2.8)	2 (5.4)	1.02 (0.23–4.50)
					
Mycosis fungoides/sezary	16	0 (0)	1 (6.7)	1 (6.3)	1.56 (0.19–12.94)
Other T cell^d^	21	1 (4.8)	0 (0)	1 (4.8)	0.84 (0.11–6.57)

a Excluded one control and 17 lymphoma cases HIV positive and eight lymphoma cases organ allograph recipients.

b OR adjusted age, sex and centre of recruitment.

c Other B-cell lymphoma includes: nine, mantle lymphoma; two, hairy cell; one, Burkitt; three, high-grade lymphoma; nine, precursor B lymphoblastic lymphoma/leukaemia; one, high-grade lymphoma.

d Other T-cell lymphoma includes: large granular lymphocytic leukaemia; two, peripheral T-cell lymphomas, unspecified; three, angioimmunoblastic T-cell lymphoma; three, angiocentric lymphoma; eight, anaplastic large-cell lymphoma CD30+; one, lymphoma T not otherwise specified, which was the only one HHV8 positive.

OR=odds ratio; HHV-8=human herpesvirus-8; CI=confidence interval, HIV=human immunodeficiency virus. NA=not applicable.

). HHV-8 seroprevalence was similar for cases and controls (OR=1.04, 95% CI=0.62–1.75). Within B-cell lymphomas, HHV-8 infection was associated with an increased risk of lymphoplasmacytic lymphoma (OR=4.47, 95% CI=1.34–14.85) and of low-grade B-cell lymphoma not otherwise specified (NOS) and lymphoma NOS (OR=5.82, 95% CI=1.07–31.73).

HHV-8 DNA was identified in nine of 69 (13.0%) HHV-8 seropositive subjects, including those HIV-infected subjects and organ recipients and in none of the 132 seronegative subjects matched by age and sex to positive subjects and randomly selected from the pool of negatives. Two subjects showed a high HHV-8 copy number, one patient with a T-cell lymphoma (copy number per 10^6^ cells=305 882), who was coinfected with HIV and one subject HIV negative with a B-cell lymphoma NOS, who had been previously diagnosed with MCD (copy number per 10^6^ cells=24 444).

## DISCUSSION

In our study, no overall differences in the HHV-8 prevalence could be found between cases and controls. However, HHV-8 was strongly associated with two subgroups, a four-fold increased risk of lymphoplasmacytic lymphoma and a five-fold increased risk of low-grade B-cell lymphoma and B-cell lymphoma NOS. The detection of HHV-8 DNA in PBMC showed that only one in seven seropositive subjects had viral DNA detectable in blood. A possible aetiologic association with HHV-8 was suspected in two subjects with a very high viral DNA copy number in PBMC. One was an HIV-positive subject with a T-cell lymphoma. This observation is in agreement with a recent case report of a PEL of T-cell origin associated with HHV-8 was suggested in an HIV-negative patient (Lechapt-Zalcman *et al*, 2001). The other subject was a B-cell lymphoma NOS with a previous diagnosis of MCD, suggesting that HHV-8 in this case is likely to play an aetiological role. This observation is in agreement with other reports where MCD has also been associated with other lymphoid neoplasms such as plasmablastic lymphoma and the recently proposed germinotropic lymphoproliferative disorder (Du *et al*, 2001) that involves a proliferation of plasmablasts.

In our data, we also observed a slight increased risk associated with HHV-8 for splenic marginal zone lymphomas. These neoplasms can also harbour plasma cells with cytoplasmatic immunoglobulins and must be considered within the range of differential diagnosis of lymphoplasmacytic lymphoma (Whitby *et al*, 1995). It has been suggested that HHV-8 could act by infecting IgM-positive naïve cells and drive these cells to differentiate into plasmablastic cells without undergoing the genetic rearrangements that take place within the germinal centre (Du *et al*, 2001). Our data are consistent with the hypothesis that HHV-8 targets B cells before they enter the germinal centre, no increased risk being observed in our study among plasma cell neoplasms that involve plasma cells at a more mature stage.

HHV-8 is not readily isolated in cell culture. HHV-8 DNA can be detected by PCR in all KS tumour biopsies. However, viral DNA is detectable by nested PCR in the blood in only half of KS patients and 10% of asymptomatically infected subjects because few infected cells are present in peripheral blood (Biggar *et al*, 2003). Serological assays are considerably more sensitive. Our second-generation K8.1 ELISA consistently had sensitivity and specificity values close to 100% (Corchero *et al*, 2001).

Our case–control study was based on hospitalised patients with high participation rates for both cases and controls. Serology was obtained at the time of study entry. A bias in our estimates could take place if the underlying HHV-8 prevalence of the control population could affect hospitalisation rates due to HHV-8-related diseases. Available data from the Spanish general population, however, indicate that the HHV-8 prevalence ranges between 6 and 8% (Gambus *et al*, 2001; de Sanjose *et al*, 2002), which is similar to the 5.3% observed in our control subjects, and also conforms to the moderate prevalence observed in Mediterranean countries. Owing to small numbers in some lymphoma categories, we cannot exclude that some of the results are due to chance.

Overall, our results suggest that HHV-8 is unlikely to contribute importantly to lymphomagenesis. The association with lymphoplasmacytic lymphoma and with low-grade lymphoma deserves further research.
